# NGS-based deep bisulfite sequencing

**DOI:** 10.1016/j.mex.2015.11.008

**Published:** 2015-11-26

**Authors:** Suman Lee, Joomyeong Kim

**Affiliations:** Department of Biological Sciences, Louisiana State University, Baton Rouge, LA 70803, USA

**Keywords:** NGS, Next-Generation-Sequencing, PCR, polymerase chain reaction, DNA methylation, Bisulfite sequencing, Epigenomes, NGS

## Abstract

We have developed an NGS-based deep bisulfite sequencing protocol for the DNA methylation analysis of genomes. This approach allows the rapid and efficient construction of NGS-ready libraries with a large number of PCR products that have been individually amplified from bisulfite-converted DNA. This approach also employs a bioinformatics strategy to sort the raw sequence reads generated from NGS platforms and subsequently to derive DNA methylation levels for individual loci. The results demonstrated that this NGS-based deep bisulfite sequencing approach provide not only DNA methylation levels but also informative DNA methylation patterns that have not been seen through other existing methods.

• This protocol provides an efficient method generating NGS-ready libraries from individually amplified PCR products.
• This protocol provides a bioinformatics strategy sorting NGS-derived raw sequence reads.
• This protocol provides deep bisulfite sequencing results that can measure DNA methylation levels and patterns of individual loci.

## Graphical abstract

**Figure:**
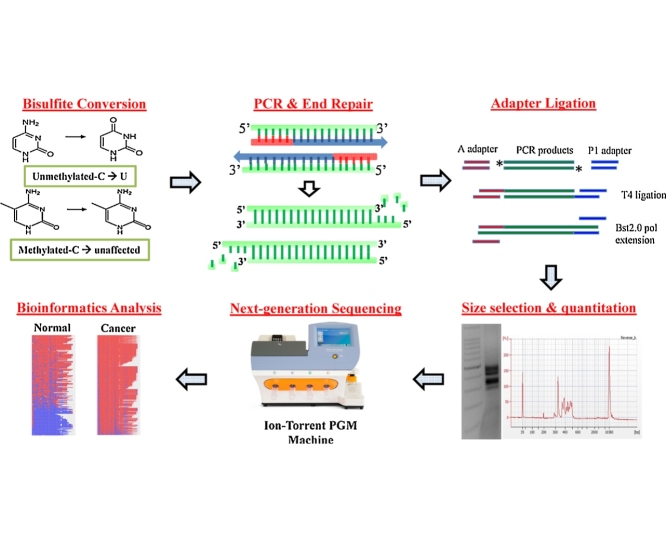

## Method details

### Overview

DNA methylation on CpG dinucleotides is as an epigenetic modification indicating the functional status of a given locus in a cell type [1]. This epigenetic mark can be determined through the bisulfite sequencing method, which involves a series of chemical reactions and subsequent sequencing of a genomic region [2]. The bisulfite sequencing method has been traditionally used for measuring the methylation levels of a small number of genomic regions in a targeted fashion. In recent years, this bisulfite sequencing method has been adopted as a genome-wide approach owing to the advancement of Next-Generation-Sequencing (NGS) platforms [3], [4]. The NGS-driven bisulfite sequencing is useful for providing a global view of epigenomes and also for identifying genomic loci with different levels of DNA methylation. For a given locus, however, the majority of the NGS-driven data are not sufficiently deep enough to report the reliable and comprehensive measurement of its DNA methylation levels due to the relatively low levels of sequencing depth. This is particularly problematic in a situation where a testing DNA is derived from a mixture of different types of cells and/or functional status.

**Fig. 1:**
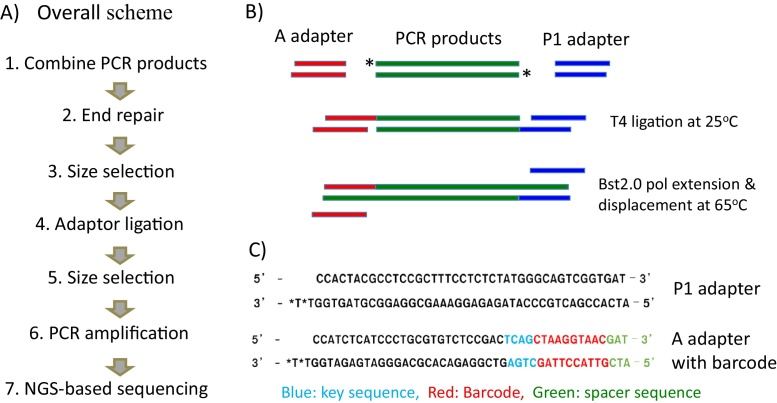
Overall scheme for NGS-based deep bisulfite sequencing. (A) The entire procedure of the NGS-based deep bisulfite sequencing protocol is shown as a flow chart. (B) The adaptor ligation step for the current protocol has adopted one strategy, in which the added PCR products and two adaptors are ligated through two stepwise incubations. Since both adaptors lack the phosphate group at their 5′-ends, the ligation reaction by T4 at 25 °C occurs between only one strand of the adaptors and the PCR products. In this case, the phosphate groups are derived from the 5′-end of the PCR products. At 65 °C, the activated Bst2.0 WarmStart polymerase extends and displaces the other unligated strand from the partially joined products. The * symbol indicates the phosphate group at the 5′-end of the end-repaired PCR products. (C) The sequences of Ion Torrent P1 and A adaptors are shown with different colors to indicate the key, barcode and spacer regions. The * symbol indicates a phosphothiate bonding between two nucleotides, which protects the duplex adaptor from being digested by the exonuclease activity of DNA polymerases.

To solve this problem, we have developed a protocol in the current study (Fig. 1A). The main objective of this protocol is NGS-based deep bisulfite sequencing of a relatively small number of genomic targets. The detailed procedure is as follows. In Step 1, this protocol pulls together a number of amplified PCR products, size-ranging from 200 to 400 base pair (bp) in length. The selection of this size range is based on the average read lengths that can be afforded by NGS platforms (here, Ion Torrent PGM2). The number of PCR products that can be grouped together as a library ranges from several hundreds to a few thousands. The DNA amount of individual PCR products needs to be normalized for different target loci to be represented evenly well among the final sequence reads from NGS runs. In Steps 2 and 3, this library of PCR products goes through the end repair reaction followed by size selection on a 2% agarose gel. In Step 4, the eluted DNA from the agarose gel is ligated to a set of duplex adaptors that are compatible with NGS platforms (in our case, Ion Torrent P1 and A adaptor) (Fig. 1B and C). During this step, multiplexing can be achieved with a set of A adaptors with different barcodes (Supplemental material 1). This ligation step has been usually problematic in many cases of library construction mainly due to the self-ligation reaction between two adaptors, which is the most dominant among all combinations of ligations. To avoid this self-ligation problem, the current protocol does not use phosphorylated adaptors. In this case, the phosphate groups are derived from the 5′-ends of the end-repaired PCR products. The current protocol also uses a mixture of T4 ligase and Bst2.0 WarmStart polymerase with two stepwise incubations, a 25-min incubation at 25 °C for the ligation reaction by T4 ligase and another 30-min incubation at 65 °C for the extension/displacement reaction by Bst2.0 WarmStart polymerase (available from NEB). The initial incubation ligates only one strand, not both, of each 5′-end of PCR products to the adaptors (Fig. 1B). Once this type of partially ligated products reaches to the 65 °C temperature, the activated Bst2.0 WarmStart polymerase initiates the extension and displacement reaction on the other strand, which has not been ligated earlier by T4 ligase. In Steps 5 and 6, the library of PCR products is then size-selected again on a 2% agarose gel, and the eluted DNA is used for PCR amplification with a set of the two primers that correspond to the two adaptors. The typical cycle numbers for this step is between 10 and 14. In Step 7, the library of amplified PCR products is used for sequencing on a desired NGS platform.

The current protocol uses a set of bioinformatic tools to process the output from NGS runs. Since the initial library is made of a number of individual PCR products, the sequencing output from a NGS run needs to be sorted as described below. First, if one of the adaptors is indexed with multiple barcodes, the sequencing output will be sorted based on the used barcodes. Second, the sequencing output is further sorted with the primer sequences for each PCR product using a custom Perl script (Supplemental material 2). Finally, the sorted sequences for each PCR product are used for calculating its DNA methylation levels with BiQ Analyzer HT [5]. A set of additional Perl scripts have been prepared to execute the process of BiQ Analyzer HT on a batch mode (Supplemental material 2).

### Reagents

**Table:** 

A adapter primer
Oligo: CCATCTCATCCCTGCGTGTC
Barcode A Adapter (Fig. 1C)
Bst2.0 WarmStart DNA polymerase (includes 10× Isothermal Amplification Buffer; New England Biolabs. Cat. No. M0538S)
DNA Clean & Concentrator™-25 (includes spin columns, DNA Binding Buffer, Wash Buffer, Elution Buffer, Zymo, Cat. No. D4005)
NEBNext End Repair Module (includes NEBNext End Repair Enzyme Mix, NEBNext End Repair Reaction Buffer; New England Biolabs, Cat. No. E6050S)
P1 Adapter (Fig. 1C)
P1 Adapter Primer
Oligo: CCACTACGCCTCCGCTTTCC
T4 DNA Ligase (includes 10× T4 DNA Ligase Reaction Buffer; New England Biolabs, Cat. No. M0202S)
TE buffer (1×, pH 8.0)
Zymoclean Gel DNA Recovery Kit (includes spin columns, ADB Buffer, Wash Buffer, Elution Buffer; Zymo, Cat. No. D4001)

### Equipment

**Table:** 

2100 Agilent Bioanalyzer System
Agarose gel (2%) and equipment for electrophoresis
Centrifuge (Benchtop centrifuge for 1.5 ml microcentrifuge tubes)
E-Gel Precast Agarose Electrophoresis System
Ion torrent PGM NGS machine
Thermocycler

### Procedure

#### Library preparation procedure

1. Combine all the PCR products that have been amplified from bisulfite-converted DNA. It is important to normalize the DNA amount of individual PCR products.
2. Prepare the end repair reaction as described below.

**Table:** 

Component	Volume
PCR product	X μl
End repair enzyme mix	5 μl
End repair reaction buffer (10×)	10 μl
H_2_O	Y μl

Total	100 μl
3. Incubate at 20 °C for 30 min in thermocycler.
4. Prepare a 2% agarose gel for size selection.
5. Load the entire DNA sample into one or two wells along with a 100-bp size marker in a separate well, and run the gel at 100 V for 40 min.
6. Excise a section of the agarose gel containing the PCR products with the desired size range, and extract the DNA from the gel using Zymoclean Gel DNA Recovery Kit. Finally, elute the DNA in 20 μl of TE.
7. Prepare the adapter ligation reaction as described below.

**Table:** 

Component	Volume
Size selection sample	17 μl
Bst2.0 warm polymerase (8000 U/ml)	1 μl
Bst2.0 polymerase buffer (10×)	4 μl
T4 DNA ligase (400,000 U/ml)	4 μl
T4 DNA ligase buffer (10x)	4 μl
P1 + A Barcode Adapter (10 pmol/μl)	10 μl

Total	40 μl
8. Incubate at 25 °C for 25 min, 65 °C for 30 min, and finally 80 °C for 20 min to stop the reaction.
9. Repeat the size selection step as described above (5–6). Elute the DNA sample in 10 μl of D.W.
10. Perform PCR to amplify the eluted DNA with a set of the two primers corresponding to P and A1 adapter: P1 adapter primer, 5′-CCACTACGCCTCCGCTTTCCTCT-3′; A adapter primer, 5′-CCATCTCATCCCTGCGTGTCTCC-3′.
11. The detailed program for PCR is shown below. The typical number of PCR cycles ranges from 10 to 14.

**Table:** 

Cycle number	Denature	Anneal	Extension
1	95 °C, 5 min		
10–14	95 °C, 30 s	68 °C, 30 s	72 °C, 45 s
1			72 °C, 10 min
12. Clean up the final PCR product using DNA Clean & Concentrator Kit.
13. Size select using E-Gel Precast Agarose Electrophoresis system.
14. Clean up the selected DNA library fragment using DNA Clean & Concentrator Kit.
15. Perform NGS-based sequencing with the prepared library.

#### Bioinformatics analysis

We used the following bioinformatics pipeline to process the raw sequence reads derived from a NGS platform. This pipeline includes several custom-made Perl scripts along with Unix-based command lines. This pipeline require two files in the text format: the first file containing a list of names for individual PCR products and the second file containing the sequences of all the primers used for the amplification of PCR products. This pipeline also requires a directory of the individual sequence files in the fasta format, which correspond to the unconverted original sequences of the PCR products. This directory is used later for calculating DNA methylation with BiQ Analayzer HT. The detailed steps are as follows:

(1) Remove raw sequence reads smaller than 100 bp in length and sort raw sequence reads into each file based on their origin of PCR products with a custom Perl script (test_sam_3.pl). The execution of this step generates a directory with a number of files containing the sorted sequence reads. Two files are usually generated for a given PCR product since this sorting process uses the sequences of the two primers that have been used to amplify the PCR product.
(2) Combine the two files that belong to a same PCR product with a custom Perl script (test_merge.pl).
(3) Execute BiQ Analyzer HT with a custom Perl script (test_biq.pl). This execution requires a tsv file containing two sets of absolute directory paths. The first set is for all of the original unconverted sequence files for PCR products, whereas the second set is for all of the sorted bisulfite sequence files. The tsv file can be prepared with a custom Perl script (test_tsv.pl).
(4) Extract the necessary information (the number of reads used, % methylation, standard deviation) from the output of BiQ Analyzer HT with a custom Perl script (test_extract.pl).

The execution of these steps will finally derive one text file containing the DNA methylation level for the entire set of PCR products, which can be imported into an excel file for further calculation and inspection. The custom Perl scripts used for this process are presented in Supplemental material 2.

## Method validation

So far, we have performed three independent trials of DNA methylation analyses using this protocol [6]. These test trials have successfully derived the DNA methylation levels of on average 80% of individual PCR products. While performing these trials, however, we have come across a couple of technical issues, which may be responsible for the observed failure, the remaining 20% of the PCR products. First, the initial DNA amounts of some of these PCR products were lower than those of the other PCR products. As a consequence, the sequence reads from these products were not as well represented as the other products in the final sequencing output. It is, therefore, very important to normalize the initial DNA amounts of individual PCR products at the early stage of library construction (Step 1 in Fig. 1A). Also, it is advisable to perform an independent protocol, such as COBRA (Combined Bisulfite Restriction Analysis) [7], before the actual bisulfite sequencing given the possibility that PCR could enrich one type of DNA sequences, such as unmethylated DNA compared to methylated DNA. Second, the size of some of sequence reads turned out to be much smaller than their predicted sizes based on the initial sizes of PCR products. According to detailed inspection, the sequences of these products were truncated at homopolymeric regions, such as poly A or T. These are typical sequences for bisulfite-converted DNA since many Cs are converted to Ts during the bisulfite conversion reaction. According to our recent study [6], similar polymeric repeats are often detected in mammalian genomes, 2 out of 12 amplified PCR products with the average length being around 300 bp in length. Although the exact cause for this truncation is currently unknown, one culprit might be the potential stalling and subsequent falloff of DNA polymerase from template DNA during the sequencing reaction of NGS. One remedy to this problem may be avoiding homopolymeric regions while selecting target regions. Also, different NGS platforms tend to have different success rates in coping with this problem, thus one should consider this aspect more carefully for future projects.

**Fig. 2:**
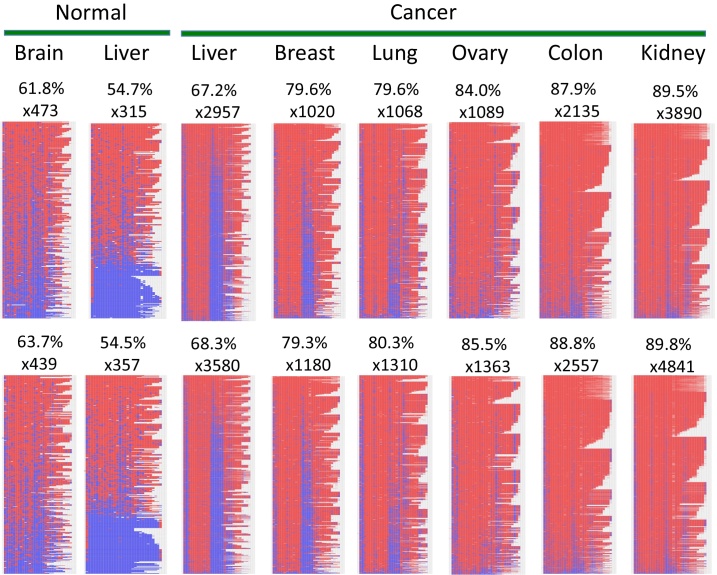
DNA methylation levels and patterns of one representative data set. The NGS-based deep bisulfite sequencing protocol was used to compare the DNA methylation levels of the promoter region of human *USP29* between the individual samples of one cancer DNA panel. This panel is comprised of the DNA isolated from two normal samples (brain and liver) and six cancer samples (liver, breast, lung, ovary, colon, kidney). Two sets of the results were derived from two independent runs of NGS. The numbers of raw sequence reads used for calculating the overall DNA methylation levels are shown underneath the name of each sample. The image composed of a large number of blue and red boxes represents the graphic summary of DNA methylation patterns. Each horizontal line represents one sequence read while each small box within this line indicates one CpG dinucleotide. The blue and red boxes indicate the unmethylated and methylated CpG sites, respectively. (For interpretation of the references to color in this figure legend, the reader is referred to the web version of this article.)

Despite these technical shortfalls, the results from the successful runs with the remaining 80% of the PCR products were very informative and insightful due to their overall deep sequencing coverage. In a particular trial measuring the DNA methylation levels of 250 PCR products, we have obtained on average 1000 sequence reads per each PCR product, which were subsequently used for measuring DNA methylation levels with BiQ Analyzer HT. We performed two independent runs of NGS sequencing to test the overall reproducibility of this protocol. One representative locus is presented in Fig. 2. Human *USP29* (Ubiquitin-specific protease 29) is an imprinted gene, yet frequently associated with several human cancers as a potential tumor suppressor [8], [9]. Thus, a set of PCR products targeting the promoter of *USP29* were amplified from a cancer DNA panel, which is made of two normal and six cancer samples. As expected, the DNA methylation levels of the two normal samples (61.8 and 63.7% for brain and 54.7 and 54.5% for liver) were lower than those from six cancer samples, which range from 67.2% to 89.8%. The higher levels of DNA methylation levels observed in the cancer samples further support the proposed tumor suppressor role for *USP29*. We observed less than 2% level difference between two independent runs (normal brain, 61.8 vs 63.7%), confirming the reproducibility of the protocol. This type of measurements can be also obtained through the other existing methods, such as Human450K array and pyrosequencing. However, the DNA methylation patterns obtained through the current protocol cannot be obtained through the other methods, although the other method, such as pyrosequencing, may provide more consistent methylation levels in a given position. As seen in Fig. 2, the two normal samples display a somewhat allele-specific DNA methylation pattern as an imprinted gene although the pattern from normal brain is mosaic. In contrast, the results from the cancer samples display unexpected DNA methylation patterns. Several patches of blue lines (representing unmethylated CpG sites) run through from bottom to top on the background of a large number of small red boxes (representing methylated CpG sites). This is particularly obvious in the two samples, liver and breast cancer samples. This indicates that several CpG sites within the promoter region of *USP29* are more resistant to DNA methylation in cancer samples. Similar patterns have also been observed in the other PCR products targeting different genomic loci (data not shown). Thus, the observed pattern appears to be an outcome of a previously unnoticed general phenomenon in mammalian epigenomes, certain CpG sites being resistant to DNA methylation. In conclusion, the current study demonstrated that the deep bisulfite sequencing by the new protocol is a very useful approach that can provide many information for mammalian epigenomes, including DNA methylation levels as well as patterns for a given locus.

## References

[1] Jaenisch R., Bird A. (2003). Epigenetic regulation of gene expression: how the genome integrates intrinsic and environmental signals. Nat. Genet..

[2] Clark S.J., Harrison J., Paul C.L., Frommer M. (1994). High sensitivity mapping of methylated cytosines. Nucleic Acids Res..

[3] Meissner A., Mikkelsen T.S., Gu H., Wernig M., Hanna J., Sivachenko A., Zhang X., Bernstein B.E., Nusbaum C., Jaffe D.B., Gnirke A., Jaenisch R., Lander E.S. (2008). Genome-scale DNA methylation maps of pluripotent and differentiated cells. Nature.

[4] Lister R., Pelizzola M., Dowen R.H., Hawkins R.D., Hon G., Tonti-Filippini J., Nery J.R., Lee L., Ye Z., Ngo Q.M., Edsall L., Antosiewicz-Bourget J., Stewart R., Ruotti V., Millar A.H., Thomson J.A., Ren B., Ecker J.R. (2009). Human DNA methylomes at base resolution show widespread epigenomic differences. Nature.

[5] Lutsik P., Feuerbach L., Arand J., Lengauer T., Walter J., Bock C. (2011). BiQ Analyzer HT: locus-specific analysis of DNA methylation by high-throughput bisulfite sequencing. Nucleic Acids Res..

[6] Kim J., Bretz C.L., Lee S. (2015). Epigenetic instability of imprinted genes in human cancers. Nucleic Acids Res..

[7] Xiong Z., Laird P.W. (1997). COBRA: a sensitive and quantitative DNA methylation assay. Nucleic Acids Res..

[8] Martin Y., Cabrera E., Amoedo H., Hernández-Pérez S., Domínguez-Kelly R., Freire R. (2015). USP29 controls the stability of checkpoint adaptor Claspin by deubiquitination. Oncogene.

[9] He H., Kim J. (2014). Regulation and function of the Peg3 imprinted domain. Genomic Inform..

