# Supplementary material for: NGS-based deep bisulfite sequencing
Source: MethodsX. 2015 Nov 26;3:1–7. doi: 10.1016/j.mex.2015.11.008 (PMC4924566; doi:10.1016/j.mex.2015.11.008)
Supplement: Supplementary file 1 [file mmc1.docx]

| Id_str | Sequence | Id_str | Sequence | Id_str | Sequence | Id_str | Sequence |
| --- | --- | --- | --- | --- | --- | --- | --- |
| IonXpress_1 | CTAAGGTAAC | IonXpress_25 | CCTGAGATAC | IonXpress_49 | TCCTAACATAAC | IonXpress_73 | TCTGCCTGTC |
| IonXpress_2 | TAAGGAGAAC | IonXpress_26 | TTACAACCTC | IonXpress_50 | CGGACAATGGC | IonXpress_74 | CGATCGGTTC |
| IonXpress_3 | AAGAGGATTC | IonXpress_27 | AACCATCCGC | IonXpress_51 | TTGAGCCTATTC | IonXpress_75 | TCAGGAATAC |
| IonXpress_4 | TACCAAGATC | IonXpress_28 | ATCCGGAATC | IonXpress_52 | CCGCATGGAAC | IonXpress_76 | CGGAAGAACCTC |
| IonXpress_5 | CAGAAGGAAC | IonXpress_29 | TCGACCACTC | IonXpress_53 | CTGGCAATCCTC | IonXpress_77 | CGAAGCGATTC |
| IonXpress_6 | CTGCAAGTTC | IonXpress_30 | CGAGGTTATC | IonXpress_54 | CCGGAGAATCGC | IonXpress_78 | CAGCCAATTCTC |
| IonXpress_7 | TTCGTGATTC | IonXpress_31 | TCCAAGCTGC | IonXpress_55 | TCCACCTCCTC | IonXpress_79 | CCTGGTTGTC |
| IonXpress_8 | TTCCGATAAC | IonXpress_32 | TCTTACACAC | IonXpress_56 | CAGCATTAATTC | IonXpress_80 | TCGAAGGCAGGC |
| IonXpress_9 | TGAGCGGAAC | IonXpress_33 | TTCTCATTGAAC | IonXpress_57 | TCTGGCAACGGC | IonXpress_81 | CCTGCCATTCGC |
| IonXpress_10 | CTGACCGAAC | IonXpress_34 | TCGCATCGTTC | IonXpress_58 | TCCTAGAACAC | IonXpress_82 | TTGGCATCTC |
| IonXpress_11 | TCCTCGAATC | IonXpress_35 | TAAGCCATTGTC | IonXpress_59 | TCCTTGATGTTC | IonXpress_83 | CTAGGACATTC |
| IonXpress_12 | TAGGTGGTTC | IonXpress_36 | AAGGAATCGTC | IonXpress_60 | TCTAGCTCTTC | IonXpress_84 | CTTCCATAAC |
| IonXpress_13 | TCTAACGGAC | IonXpress_37 | CTTGAGAATGTC | IonXpress_61 | TCACTCGGATC | IonXpress_85 | CCAGCCTCAAC |
| IonXpress_14 | TTGGAGTGTC | IonXpress_38 | TGGAGGACGGAC | IonXpress_62 | TTCCTGCTTCAC | IonXpress_86 | CTTGGTTATTC |
| IonXpress_15 | TCTAGAGGTC | IonXpress_39 | TAACAATCGGC | IonXpress_63 | CCTTAGAGTTC | IonXpress_87 | TTGGCTGGAC |
| IonXpress_16 | TCTGGATGAC | IonXpress_40 | CTGACATAATC | IonXpress_64 | CTGAGTTCCGAC | IonXpress_88 | CCGAACACTTC |
| IonXpress_17 | TCTATTCGTC | IonXpress_41 | TTCCACTTCGC | IonXpress_65 | TCCTGGCACATC | IonXpress_89 | TCCTGAATCTC |
| IonXpress_18 | AGGCAATTGC | IonXpress_42 | AGCACGAATC | IonXpress_66 | CCGCAATCATC | IonXpress_90 | CTAACCACGGC |
| IonXpress_19 | TTAGTCGGAC | IonXpress_43 | CTTGACACCGC | IonXpress_67 | TTCCTACCAGTC | IonXpress_91 | CGGAAGGATGC |
| IonXpress_20 | CAGATCCATC | IonXpress_44 | TTGGAGGCCAGC | IonXpress_68 | TCAAGAAGTTC | IonXpress_92 | CTAGGAACCGC |
| IonXpress_21 | TCGCAATTAC | IonXpress_45 | TGGAGCTTCCTC | IonXpress_69 | TTCAATTGGC | IonXpress_93 | CTTGTCCAATC |
| IonXpress_22 | TTCGAGACGC | IonXpress_46 | TCAGTCCGAAC | IonXpress_70 | CCTACTGGTC | IonXpress_94 | TCCGACAAGC |
| IonXpress_23 | TGCCACGAAC | IonXpress_47 | TAAGGCAACCAC | IonXpress_71 | TGAGGCTCCGAC | IonXpress_95 | CGGACAGATC |
| IonXpress_24 | AACCTCATTC | IonXpress_48 | TTCTAAGAGAC | IonXpress_72 | CGAAGGCCACAC | IonXpress_96 | TTAAGCGGTC |
